# Experimental Study on the Slip Behaviour of Stainless Steel High-Strength Bolted Connections with a New Surface Treatment

**DOI:** 10.3390/ma15165672

**Published:** 2022-08-18

**Authors:** Tianxiong Zhang, Yidu Bu, Yuanqing Wang, Zhihua Chen, Wei He

**Affiliations:** 1 School of Civil Engineering, Tianjin University, Tianjin 300072, China; 2 Department of Civil Engineering, Beijing University of Technology, Beijing 100124, China; 3 School of Civil Engineering, Tsinghua University, Beijing 100084, China; 4 The Institute of High Energy Physics, Chinese Academy of Sciences, Beijing 100049, China

**Keywords:** stainless steel high-strength bolted connection, faying surface treatment method, slip factor, preload, test

## Abstract

The conventional surface treatments on stainless steel plates yield slip factors less than the required value for a friction grip, which hinders the application of stainless steel high-strength bolts. The slip factor and the treatment process of stainless steel surfaces are not clearly specified in most of the existing design codes. Existing studies also show different slip factors from similarly treated surfaces. In this paper, a new surface treatment is proposed: the two touching surfaces of the bolted connection are grit-blasted on one side and coated with high-velocity oxygen fuel (HVOF) on the other side. The slip behaviour of the new method was compared with four conventional methods. The roughness and hardness of different surfaces were measured prior to a total of 30 slip tests where the slip factors of multiple stainless steel surfaces were tested. It was shown that the new proposed surface treatment method can yield a slip factor of 0.61, much higher than the conventional surface treatments, meeting the requirements as a slip-resistant connection. The preload relaxation of stainless steel high-strength bolts was also monitored and compared to standard grade 10.9 high-strength bolts. We found that the preload relaxation in stainless steel high-strength bolts is negligible.

## 1. Introduction

Stainless steel is receiving more attention among architects and structural engineers due to its high resistance to corrosion. With the increase in stainless steel production and the emergence of design specifications all over the world, stainless steel has gradually become an important building material, especially in special environments. Existing design guidance, such as EN 1993-1-4 [1], Design guide-27 [2], AS/NZS 4673:2001 [3], SEI/ASCE-8-02 [4], and CECS 410:2015 [5], do not specify a stainless steel high-strength bolted connection design due to insufficient research. A notable exception is the recommendation of stainless steel high-strength bolts in a slip-resistant connection with certain surface treatment in the Japanese standard [6]. On the other hand, the specifications of high-strength bolt connections such as EN 1090-2 [7] or RCSC [8] only consider carbon steel high-strength bolts. Due to the absence of stainless-steel high-strength bolts in all design codes, any use of preloaded stainless steel high-strength bolts must be tested. Stainless steel is also considered to be more prone to preload loss at room temperature than carbon steel. All of these factors have withheld the application of stainless steel high-strength bolts and the application of stainless steel structures in seismic areas.

There are a few existing studies of stainless steel slip-resistant connections: Wang et al. [9] measured the slip factor for the austenitic 316 stainless steel plate with wire drawing, grit blasting, and scoring surface treatments, and found that the slip factors of those treatments were lower than 0.21. Stranghöner et al. [10,11] conducted slip factor tests on different types of stainless steel and compared four surface treatments (untreated, grit-blasted, shot-blasted, and grit-blasted surface with an aluminium spray metalized coating). The obtained slip factor, though from the surface treatment with the same name, is much higher than that from Ref. [9]. Zheng et al. [12] conducted a comprehensive study on stainless steel high-strength bolted connections, where slip factors of different surface treatments were investigated. Both conventional treatment and coating treatment were studied, and an equation was proposed showing that the roughness was positively correlated with the slip factor. The slip factors of a stainless steel surface and of other materials, namely aluminium and GFRP, have been studied by Zhang et al. [13] and Peng et al. [14], respectively. Existing studies show that the stainless steel surface has a relative low slip factor, and additional surface treatment to increase the roughness can increase the slip factor only to some extent. This paper proposes a new surface treatment method. The two touching surfaces of a stainless steel high-strength bolted connection are grit-blasted on one side and high-velocity oxygen fuel (HVOF)-coated on the other side. Different from the treatment methods mentioned in the literature, this new method uses a coarse virgin plate on one side and a coated plate on the other side, changing the load mechanism in the slipping process and obtaining a higher slip factor with similar roughness. The friction surface treatment method proposed in this paper has been used in the bolted connections in the main structure of the Jiangmen neutrino detector [15]. Additionally, as the preload losses of stainless steel bolts were prone to be questioned due to the visco-plastic deformation of the stainless steel material and the limited numbers of studies [16,17] the preload relaxation of stainless steel bolts with the proposed surface treatment was monitored.

In this paper, the slip behaviour of stainless steel high-strength bolted connections was investigated. The roughness and hardness of the surface under four traditional surface treatments and the proposed surface treatment were measured. A total of 30 slip factor tests were carried out to measure the slip factor for each surface treatment. In addition to the static tests, creep tests were also carried out on the stainless steel bolted connections using the new surface treatment method. The slip factors and roughness values from the existing literature were collected and compared. Finally, the preload relaxation of the Class 10.9 high-strength bolts and stainless steel high-strength bolts was monitored.

## 2. New Surface Treatment Method and Stainless Steel High-Strength Bolts

**Figure 1:**
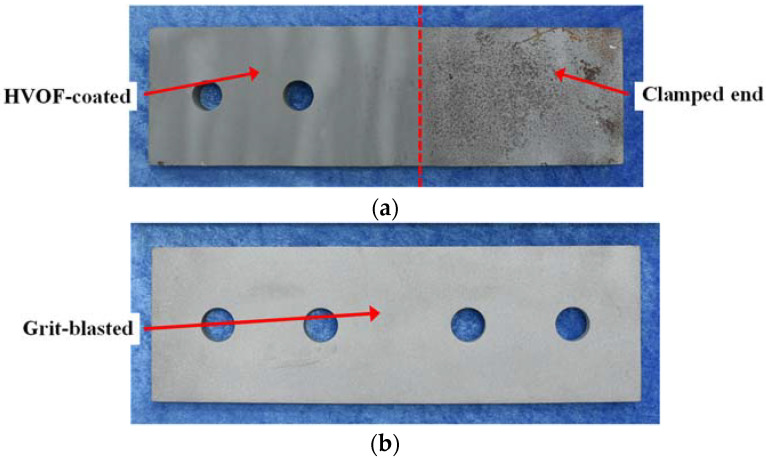
Two sides of the new surface treatment method. (**a**) Inner plate (HVOF-coated). (**b**) Cover plate (grit-blasted).

Grit blasting or shot blasting uses irregularly shaped or spherical abrasive particles to give the surface a certain roughness. There are also some new surface treatments to improve the roughness through additive spraying, such as the aluminium spray metalized coating used in Ref. [11] and the stainless steel powder and inorganic zinc-rich coating used in Ref. [12]. In this study, a new method is proposed: the grit blasting–high-velocity oxygen fuel (HVOF) coating method. This new treatment adopts different treatment methods for the two contact surfaces, where for one side the surface is treated with additive coating (HVOF-coated with stainless steel powder), while for the other side the surface is treated with abrasive particles (grit-blasted). The two surfaces are shown in Figure 1.

**Figure 2:**
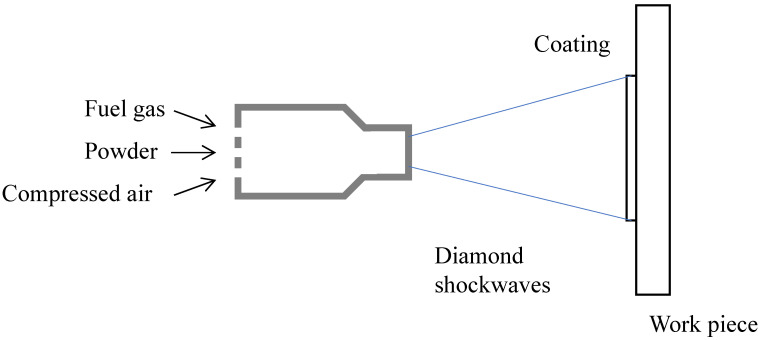
Schematic drawing of the HVOF thermal spraying system.

The surface treatment were carried out in Huadun New Material Technology Co., Ltd. in Ningbo, China. For grit blasting, brown corundum sand was used as the abrasive particle. The plate was grit-blasted with compressed air between 0.6 to 0.8 MPa, a spraying angle between 15 to 30 degrees, a spraying distance between 100 to 300 mm, and an abrasive particle size between 15 to 45 mesh. For HVOF coating, 304 stainless steel powder was dusted using a WokaStar-610 spray gun on the pre-treated surface to form the coating with a thickness between 150 to 200 µm. The adhesion of the coating should be more than 50 MPa, and the coating surface roughness Ra should not be less than 1.2 µm. The porosity of the coating should be less than 1.5%. Figure 2 shows the principle of HVOF thermal spraying [18].

**Figure 3:**
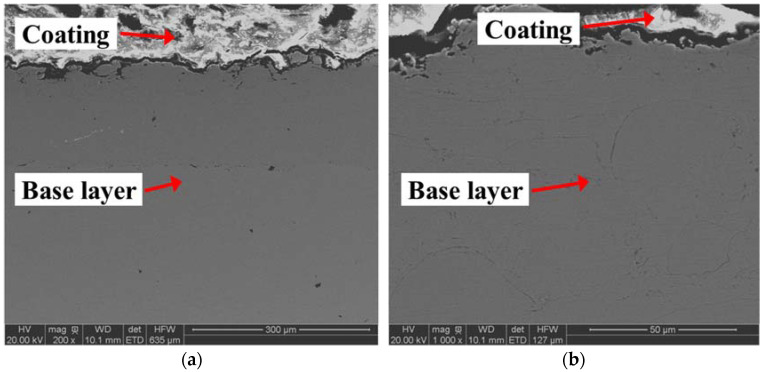
HVOF-coated plate cross-section morphology: (**a**) 200 times, (**b**) 1000 times.

The HVOF-coated plate was scanned by an electron microscope (Figure 3). The dark gray part is the base layer of the stainless steel plate and the white part is the coating. The thickness of the coating in Figure 3a was measured with the software of the microscope. The average thickness of 40 selected points was 166.9 ± 47.4 μm. The stainless steel surface after spraying was flat and uniform. The white part in Figure 3b shows that the coating was very dense and it was difficult to see the existence of pores.

The stainless steel high-strength bolts studied herein were fabricated according to conventional Class 10.9 M20 bolts, as they were not included in the relevant specification. The bolts were made from S51740 (05Cr17Ni4Cu4Nb) precipitation hardening stainless steel and were heat-treated. The bolt dimension was the same as conventional high-strength bolts, following the requirements for standard high-strength bolts in GB/T 1228-2006 [19] and GB/T 1229-2006 [20]. The mechanical properties of these stainless steel high-strength bolts have been investigated in another study [21]. The obtained average ultimate strength *σ*_u_ was 1147 MPa. The mechanical features of the stainless steel high-strength bolts were similar to those of standard Class 10.9 bolts, which meets the requirements from ISO 898-1:2013 [22].

## 3. Experimental Investigations on Slip Factors

### 3.1. Specimens Design

**Figure 4:**
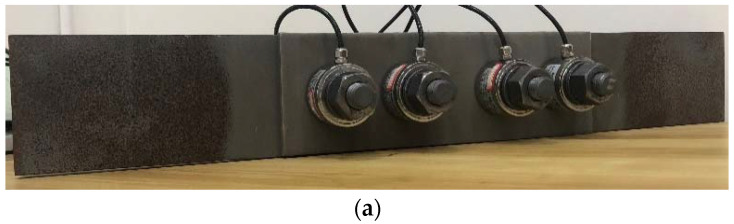

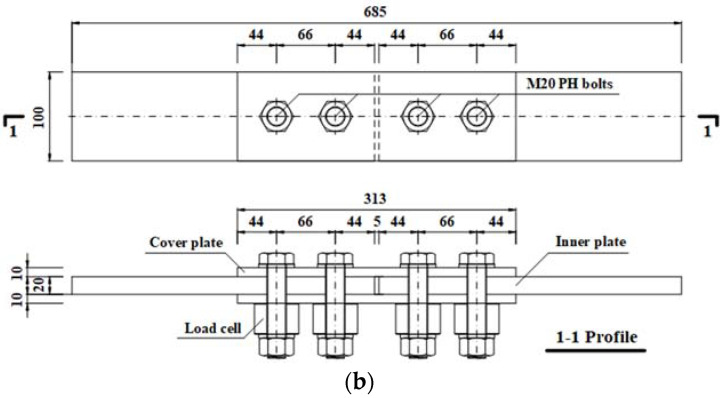
Slip factor test specimens. (**a**) Photo. (**b**) Schematic drawing.

The specimens were designed according to the requirements in Ref. [23]. The specimen was composed of a pair of inner plates and a pair of cover plates, which were connected by four stainless steel high-strength bolts (Figure 4). The inner plates were 100 mm-wide and 20 mm-thick, and the cover plates were 100 mm-wide and 10 mm-thick. The plates were made of austenitic S31608 stainless steel. The diameter of the bolt hole was 22 mm for M20 bolts.

In this paper, five different surface treatments were used on the surface of bolted connections with stainless steel high-strength bolts. The corresponding slip factors of different treatment methods were obtained through experiments. Four conventional treatments for carbon steel structures were investigated, including polishing, brushing, shot blasting, and grit blasting. The proposed treatment, grit blasting–HVOF coating, was also investigated. In total, 30 specimens were designed (Table 1).

### 3.2. Roughness and Hardness Measurements

For each friction surface treatment, the roughness and hardness of the surface were measured prior to the slip test. It was obtained according to the requirements in GB/T 10610-2009 [24] with a roughness tester TR200. Since the actual plate surface roughness was not evenly distributed, an average of five test points was taken along the hole circumference of each plate. After removing the maximum and minimum values from the readings of the five test points, the average of the remaining three test points was taken as surface roughness Ra. The measured roughness is listed in Table 2. For the new surface treatment method, only the roughness of the HVOF coating was listed, while the surface roughness of the grit-blasted side was similar to the conventional grit blasting method and was not separately listed.

The HRB hardness of stainless steel plates with different treatments was measured using the Rockwell hardness test method [25]. For each surface treatment, one plate was selected. Three measurements were made around the bolt hole. The average measured hardness is listed in Table 2. Note that only the hardness of the HVOF coating is listed.

### 3.3. Static Tests

**Figure 5:**
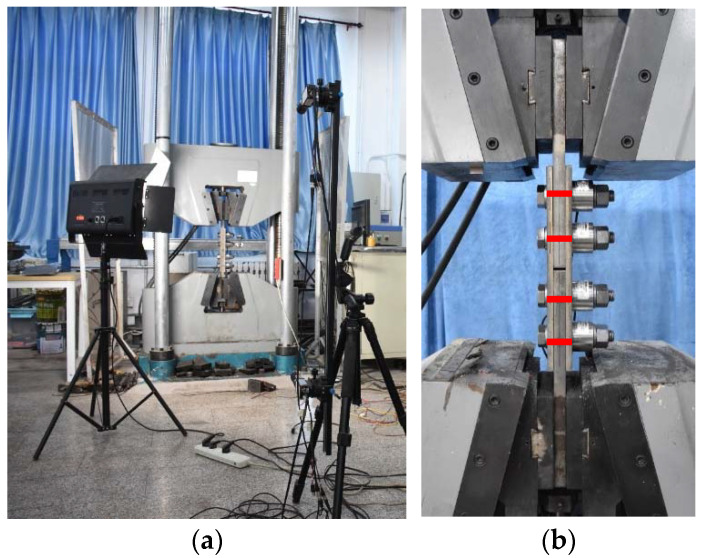
Slip factor test setup. (**a**) Test device and DIC. (**b**) Test specimen.

All specimens were tested following the relevant provisions in Appendix G of EN1090-2 [7]. The preload level of the stainless steel high-strength bolts was measured with load cells. The nominal preload P was 170 kN for M20 10.9 stainless steel high-strength bolts. After the bolt preload was applied, auxiliary lines were drawn along the thickness of the specimen at the axial line of the bolts, as indicated by the red line in Figure 5b. During the test, the relative displacement of the auxiliary line on the plates was measured by digital image correlation (DIC). A loading rate of 1 mm/min was applied with a 100-ton universal testing machine (Figure 5).

**Figure 6:**
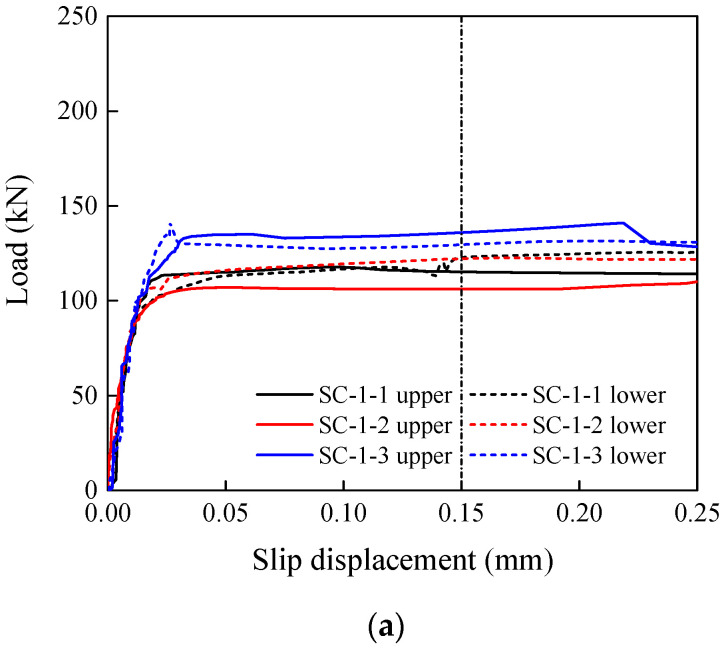

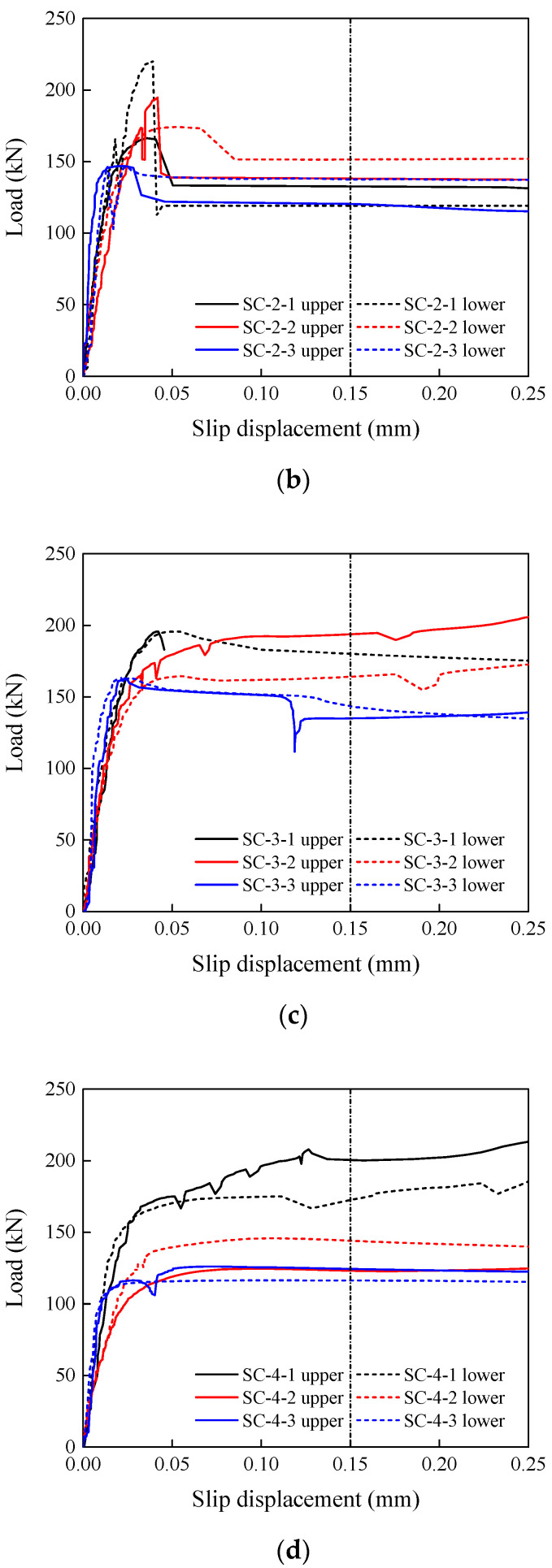

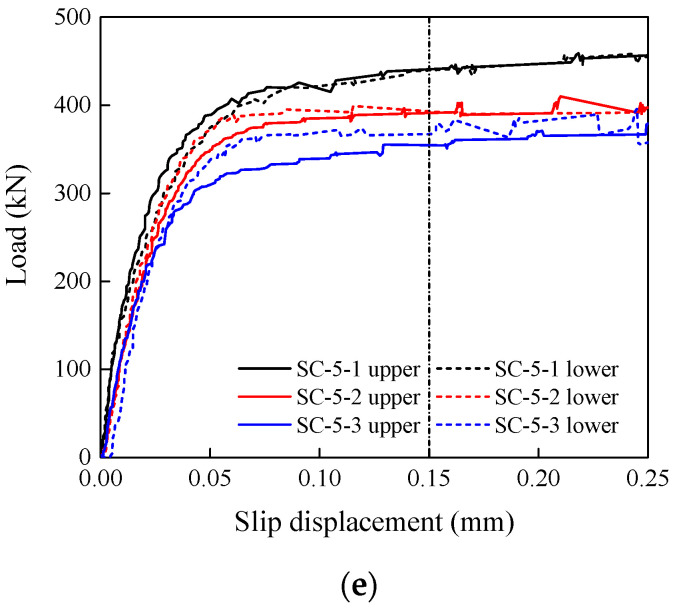
Typical load–displacement curves of slip factor tests. (**a**) Polishing. (**b**) Brushing. (**c**) Grit blasting. (**d**) Shot blasting. (**e**) Grit blasting–HVOF coating.

**Figure 7:**
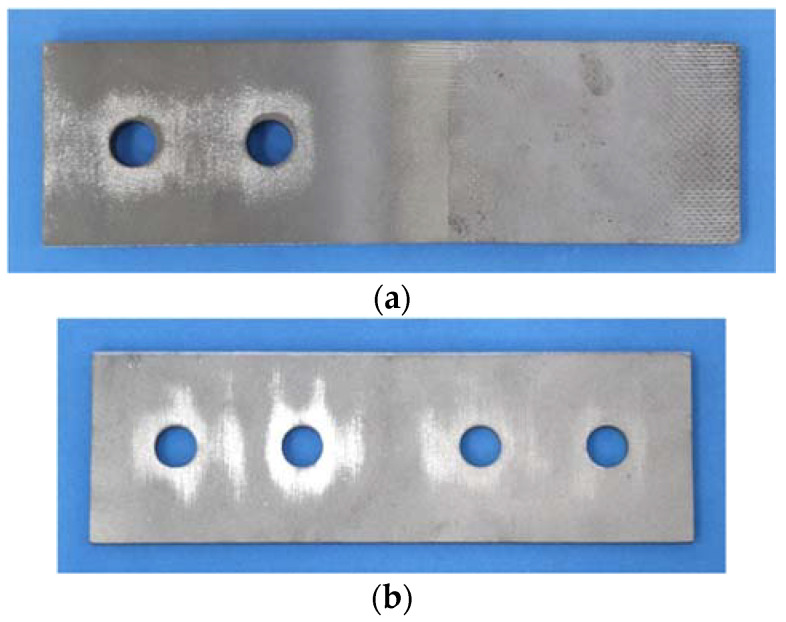
Abrasion around the bolt hole after the tests. (**a**) Inner plate (HVOF-coated). (**b**) Cover plate (grit-blasted).

Typical load–slip displacement curves obtained from the tests are shown in Figure 6. The slip displacement was the relative slip between the inner plate and the cover plate captured by the DIC camera, while the load was recorded by the universal testing machine. The upper and lower curves were plotted with the average relative slip displacement from the top or bottom auxiliary lines, respectively. The slip load N_v_ was the peak load before or at the 0.15 mm slip criterion, taking the smaller value from the upper and lower curves. Zheng et al. [12] used the displacement of the cross beam as the slip displacement, which includes the elastic elongation of the plates, the possible creep of the inner plate, and the possible sliding of the clamping section. To solve this problem, a DIC camera was used to directly monitor the relative displacement of the plates and describe the slip more accurately. For some of the specimens, a sound can be heard at the moment of the slip. Figure 7 shows that specimens with a larger slip factor tend to make a sound when slipping. The tested specimens show an abrasive area near the bolt holes, which is more obvious on the slipping end.

The slip factor can be determined from the following equation:

(1) $$\mu =N_{v}/(n_{f}\sum_{i=1}^{m}P_{i})$$

where *N*_v_ is the slip load; n_f_ is equal to 2, representing the number of friction surfaces; *m* is equal to 2, representing the number of bolts on one end of the specimen; and *P_i_* is the preload of a single bolt, which was recorded by the load cell. The test results, using conventional steel surface treatments and the new treatment proposed in this paper, are shown in Table 2.

It can be seen that the conventional steel structure surface treatment can improve the slip factor of the stainless steel plate up to 0.26, but this is not high enough to meet the requirements of high-strength bolt connections [7]. The proposed treatment (grit blasting–HVOF coating) yields an average slip factor of stainless steel plates of 0.61 with a standard deviation of 0.06. With the proposed method, by coating only one surface with the HVOF stainless steel powder, the slip factor is significantly enhanced. It is an economical and rapid stainless steel surface treatment.

**Figure 8:**
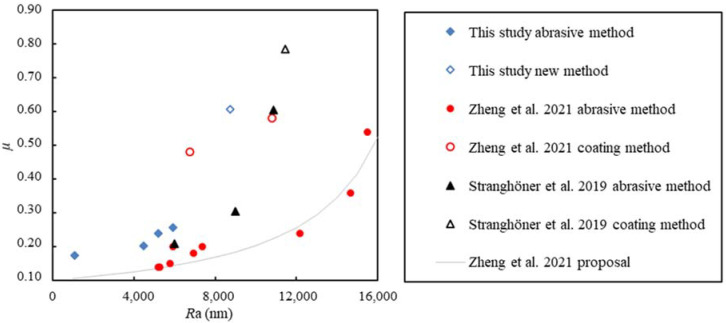
Relationship between slip factor and roughness [10,12].

It is generally believed that the slip factor increases with increasing roughness. Zheng et al. [12] proposed a nonlinear formula to express this positive correlation between the roughness and the slip factor. Figure 8 shows the roughness and slip factors obtained in this paper, as well as in Stranghöner et al. [10] and Zheng et al. [12], together with the nonlinear formula proposed by Zheng et al. [12]. It can be seen that the specimens with the coating process (presented as hollow data points) successfully improved the slip factor. This is because with the coating treatment, the contact is between the coating and the treated plate, changing the slip mechanism. As in traditional tribology, the friction force comes from the roughness of the contact surface, and the energy is largely consumed by the mutual engagement, contact, and wear between the uneven surfaces. The proposed grit blasting–HVOF coating treatment has one abrasive surface and one coated surface. This half-abrasive–half-coating method can enhance the mutual engagement between the two surfaces and increase the slip factor effectively.

The curve proposed by Zheng et al. [12] reflects that the slip factor is positively correlated to the toughness. However, existing data do not fall on the prediction curve. It can be noted that the roughness of the untreated “as rolled” surface in Stranghöner et al. [10] is larger than the roughness of some treated surface measured from this paper and Ref. [12], showing large discrepancies in the products from different manufacturers.

### 3.4. Creep Tests

**Figure 9:**
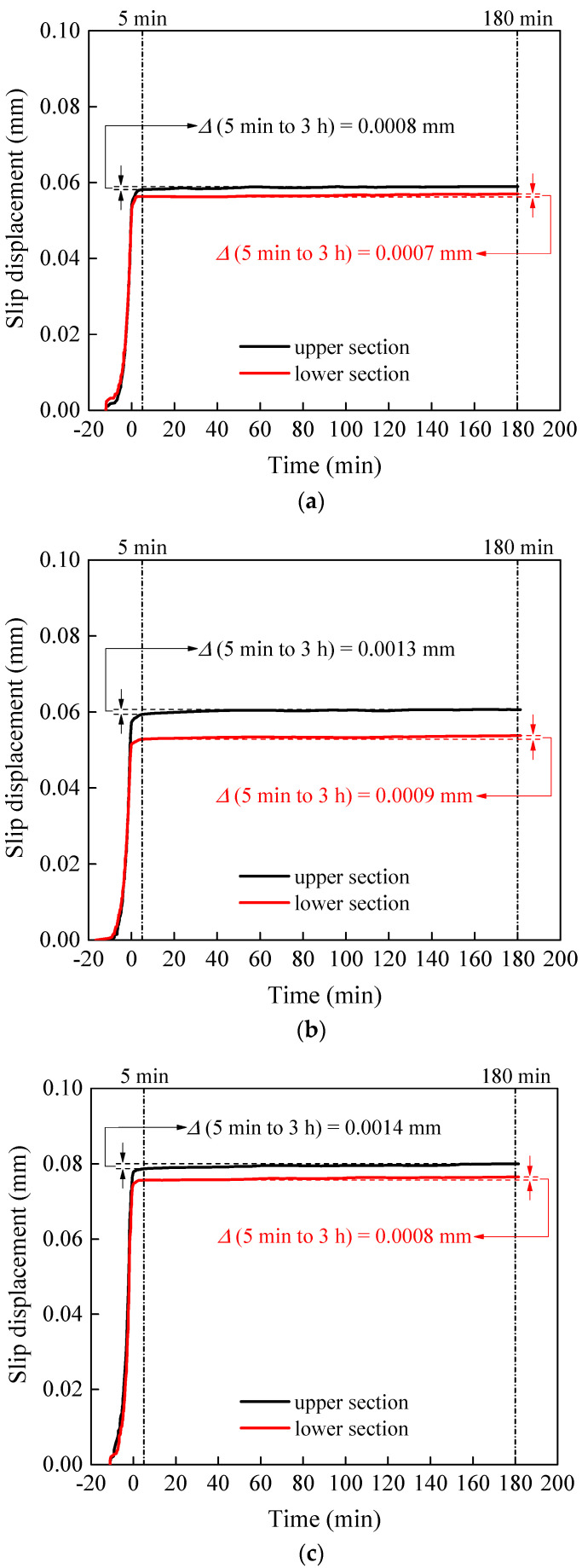
Creep test curves. (**a**) Creep test 1. (**b**) Creep test 2. (**c**) Creep test 3.

The creep behaviour of stainless steel high-strength bolts after tightening was studied through three groups of slip-resistant connection specimens. The specimens were treated with the proposed surface treatment (grit blasting–HVOF coating) and tested following the specification Annex G of EN 1090-2 [7]. The test specimen and test setup were consistent with the static test. Similar to the static test, the preload in the bolts was recorded by load cells. However, for the creep tests, the core plates were loaded with 90% of the average sliding load, *N_v_*, of the static slip factor test and held for 180 min. The slip distance–time curves are shown in Figure 9. The creep behaviour was considered negligible when the slip difference between 5 min and 3 h was less than 0.002 mm.

## 4. Preload Relaxation Monitoring

**Figure 10:**
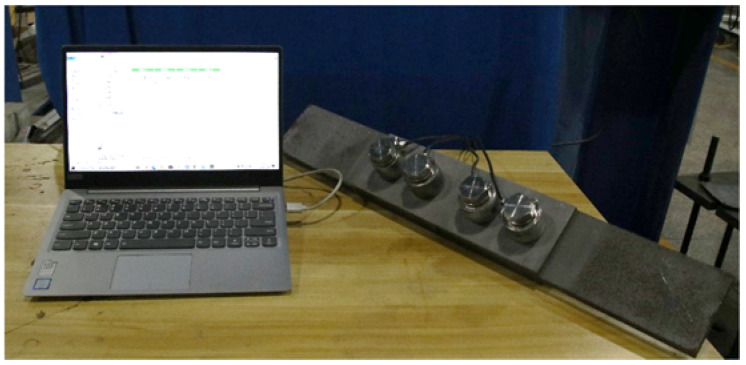
Preload relaxation test setup.

**Figure 11:**
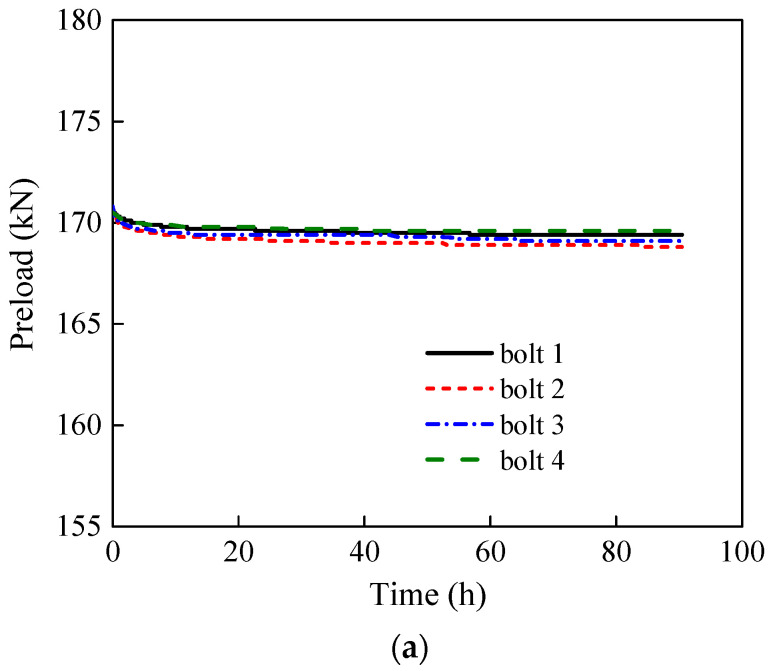

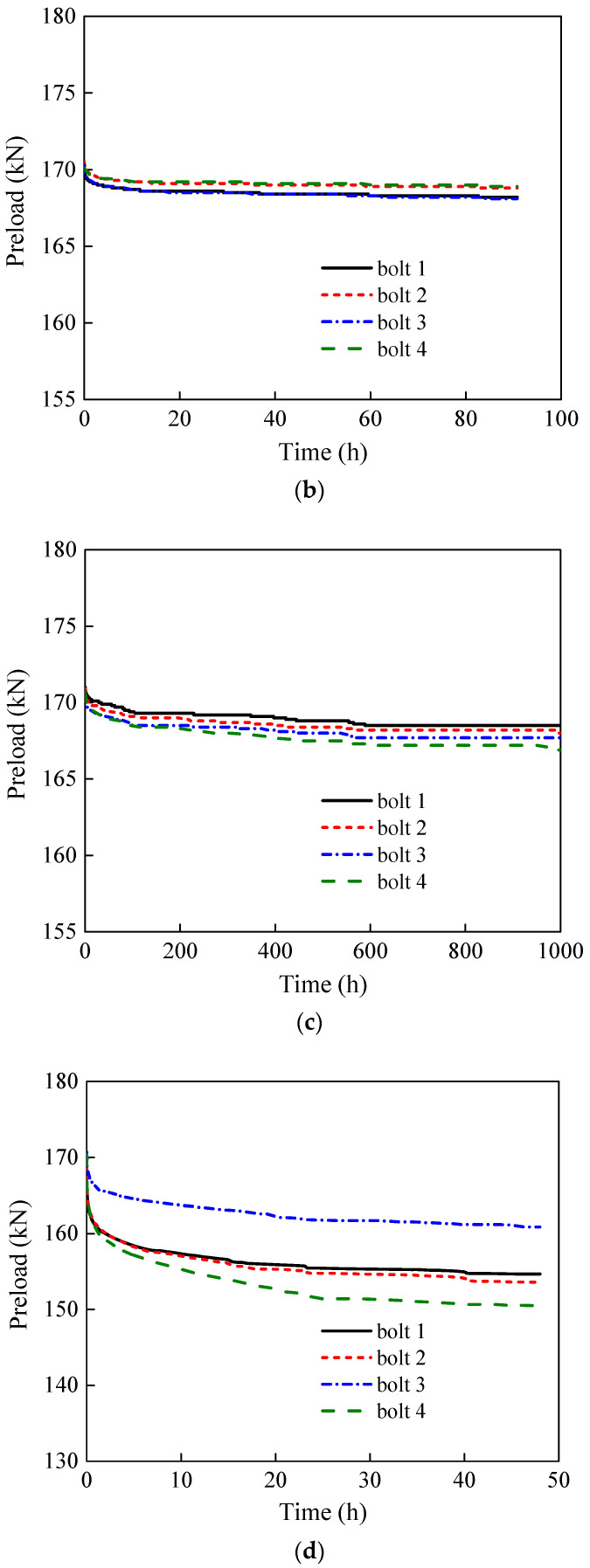
Preload relaxation curves. (**a**) Stainless steel high-strength bolt Group 1. (**b**) Stainless steel high-strength bolt Group 2. (**c**) Stainless steel high-strength bolt Group 3. (**d**) Class 10.9 carbon steel high-strength bolts.

The relaxation behaviour of preloaded stainless steel bolt components was investigated by monitoring and comparing the preload level of stainless steel high-strength bolts and Class 10.9 high-strength bolts. A total of four groups were investigated, where Group 1 and Group 2 were two repetitive groups of stainless-steel high-strength bolts with 90 h of monitoring time, Group 3 was stainless steel bolts with 1000 h of monitoring time, and Group 4 was Class 10.9 high-strength bolts with 48 h of monitoring time. The test was set up following the provisions in EN ISO 4017 [25]. A set of four bolts in each group, the same as in the slip tests, was used, and the preload in each bolt was monitored, as shown in Figure 10. The proposed grit blasting–HVOF coating surface treatment was used on the specimens for preload monitoring. A load cell was used to measure and record the preload. For both types of bolts, a 170 kN preload was applied, which is 10% higher than the design preload value of M20 high-strength bolts [26,27]. The monitored preload relaxation curves are shown in Figure 11. The preload relaxation is shown in Table 3. After the bolts were tightened, the preload loss started immediately and the preload loss rate decreased over time. At the beginning of the test, the highest rate can be observed.

It can be seen from Table 3 that within 48 h after tightening the Class 10.9 high-strength bolts, the preload of the bolts gradually decreased with time. The preload decreased in the initial monitoring period faster, and the decrease rate became slower afterwards. After around 40 h, the change was relatively smooth. About 90% of the preload loss happened in the first 20 h. For stainless steel high-strength bolts, the average preload losses 90 h after tightening for Groups 1, 2, and 3 were 1.5 kN, 1.8 kN, and 1.8 kN, respectively. Group 3 showed an average preload loss of only 3 kN 1000 h after tightening. Apart from the negligible total preload loss, the preload level of stainless steel high-strength bolts tends to stabilise after 48 h. The average preload losses at 48 h after tightening for Groups 1, 2, and 3 were 1.4 kN, 1.5 kN, and 1.4 kN, respectively, accounting for 93%, 87%, and 87% of their total preload loss.

## 5. Conclusions

In this study, a new friction surface treatment for stainless steel slip-resistant connections was proposed. The two surfaces connected by stainless steel high-strength bolts were grit-blasted on one side and HVOF-coated on the other side. The slip factor of the new method was tested and compared with four conventional surface treatment methods through slip tests. The roughness and hardness of the friction surface under five surface treatments were measured, and both static and creep slip tests were carried out. It was shown that the slip factor of stainless steel plates obtained by the conventional friction surface treatments (polishing, brushing, shot blasting, and girt blasting) fail to meet the requirements of high-strength bolt connections. The stainless steel friction surface treated by the proposed method (grit blasting HVOF coating) was significantly improved. The slip specimen with the proposed surface treatment passed the creep test and can be recommended for engineering practice. The surface roughness and slip factors obtained from the literature were also compared in this paper. Although there was a positive correlation between the roughness and the slip factor, it was found that, due to a different slip mechanism, plates with the same roughness had different slip factors. The proposed half abrasive-half coating method was shown to enhance the mutual engagement, contact, and wear between uneven surfaces, thus effectively increasing the slip behaviour. The preload loss of stainless steel as well as Class 10.9 high-strength bolts were also monitored, and the preload loss of stainless steel high-strength bolts was found to be negligible.

## Figures and Tables

**Table 1:** Summary of slip factor test specimens.

Surface Treatment	Number of Specimens
Polishing	3
Brushing	3
Grit blasting	3
Shot blasting	3
Grit blasting–HVOF coating	18

**Table 2:** Slip factor test results.

Surface Treatment	Specimen ID	Pi (kN)		Slip Load Nv (kN)	Slip Factor *µ*	Average Slip Factor *µ*	Roughness Ra (µm)	Hardness (HRB)	Sound
		P1	P2						
Polishing	SC-1-1	171.1	170.8	117.8	0.17	0.18	1.10	98.5	No
	SC-1-2	171.5	170.3	106.9	0.16				No
	SC-1-3	172.6	171.8	135.7	0.20				No
Brushing	SC-2-1	168.7	168.2	166.4	0.25	0.24	5.22	94.3	No
	SC-2-2	170.7	170.3	174.3	0.26				No
	SC-2-3	169.9	170.2	146.9	0.22				No
Grit blasting	SC-3-1	169.4	170.1	195.7	0.29	0.26	5.93	93.6	No
	SC-3-2	168.8	169.5	164.8	0.24				No
	SC-3-3	169.5	170.2	163.2	0.24				No
Shot blasting	SC-4-1	171.8	169.3	175.5	0.26	0.20	4.46	97.0	No
	SC-4-2	171.4	171.5	124.6	0.18				No
	SC-4-3	170.6	169.3	116.5	0.17				No
Grit blasting–HVOF coating	SC-5-1	171.6	169.9	442.0	0.65	0.61	8.75	95.0	Yes
	SC-5-2	170.5	170.3	397.1	0.58				Yes
	SC-5-3	171.1	170.5	355.2	0.52				Yes
	SC-5-4	170.2	169.4	378.9	0.56				Yes
	SC-5-5	170.0	169.9	383.2	0.56				Yes
	SC-5-6	170.2	171.8	380.2	0.56				Yes
	SC-5-7	172.4	170.8	449.6	0.66				Yes
	SC-5-8	171.3	170.6	461.4	0.67				Yes
	SC-5-9	170.5	172.1	417.9	0.61				Yes
	SC-5-10	171.4	171.1	391.8	0.57				Yes
	SC-5-11	170.9	170.2	395.0	0.58				Yes
	SC-5-12	169.2	170.4	336.5	0.50				Yes
	SC-5-13	170.5	169.1	393.0	0.58				Yes
	SC-5-14	172.2	170.1	467.7	0.68				Yes
	SC-5-15	171.6	168.7	458.5	0.67				Yes
	SC-5-16	170.7	170.9	451.7	0.66				Yes
	SC-5-17	171.2	169.7	450.8	0.66				Yes
	SC-5-18	170.9	170.4	450.3	0.66				Yes

**Table 3:** Results of preload relaxation.

Bolt Type	Bolt Number	Preload (kN)					
		0 h	2 h	24 h	48 h	90 h	1000 h
SS 10.9 Group 1	1	170.7	170.1	169.6	169.5	169.4	-
	2	170.8	169.8	169.2	169.0	168.8	-
	3	170.8	169.9	169.4	169.3	169.1	-
	4	170.5	170.1	169.8	169.6	169.6	-
SS 10.9 Group 2	1	170.2	169.1	168.6	168.4	168.2	-
	2	170.5	169.6	169.1	169.0	168.8	-
	3	170.3	169.2	168.5	168.4	168.1	-
	4	170.1	169.5	169.2	169.1	168.9	-
SS 10.9 Group 3	1	171.0	170.6	170.1	169.9	169.5	168.5
	2	170.9	170.4	169.8	169.5	169.2	168.0
	3	170.5	170.0	169.3	169.1	168.7	167.7
	4	170.7	170.1	169.4	169.1	168.6	166.9
Class 10.9	1	170.6	160.0	155.5	154.7	-	-
	2	170.5	160.1	154.8	153.6	-	-
	3	170.7	165.5	161.8	160.9	-	-
	4	170.4	159.2	151.6	150.5	-	-

## Data Availability

Not applicable.
